# Fluorescent Labeling of Newborn Dentate Granule Cells in GAD67-GFP Transgenic Mice: A Genetic Tool for the Study of Adult Neurogenesis

**DOI:** 10.1371/journal.pone.0012506

**Published:** 2010-09-02

**Authors:** Shengli Zhao, Yang Zhou, Jimmy Gross, Pei Miao, Li Qiu, Dongqing Wang, Qian Chen, Guoping Feng

**Affiliations:** 1 Department of Neurobiology, Duke University Medical Center, Durham, North Carolina, United States of America; 2 Department of Pathology, Duke University Medical Center, Durham, North Carolina, United States of America; 3 Institute of Neuroscience and State Key Laboratory of Neuroscience, Shanghai Institutes for Biological Sciences, Chinese Academy of Sciences, Shanghai, China; Institut de la Vision, France

## Abstract

Neurogenesis in the adult hippocampus is an important form of structural plasticity in the brain. Here we report a line of BAC transgenic mice (GAD67-GFP mice) that selectively and transitorily express GFP in newborn dentate granule cells of the adult hippocampus. These GFP^+^ cells show a high degree of colocalization with BrdU-labeled nuclei one week after BrdU injection and express the newborn neuron marker doublecortin and PSA-NCAM. Compared to mature dentate granule cells, these newborn neurons show immature morphological features: dendritic beading, fewer dendritic branches and spines. These GFP^+^ newborn neurons also show immature electrophysiological properties: higher input resistance, more depolarized resting membrane potentials, small and non-typical action potentials. The bright labeling of newborn neurons with GFP makes it possible to visualize the details of dendrites, which reach the outer edge of the molecular layer, and their axon (mossy fiber) terminals, which project to the CA3 region where they form synaptic boutons. GFP expression covers the whole developmental stage of newborn neurons, beginning within the first week of cell division and disappearing as newborn neurons mature, about 4 weeks postmitotic. Thus, the GAD67-GFP transgenic mice provide a useful genetic tool for studying the development and regulation of newborn dentate granule cells.

## Introduction

In the dentate gyrus of the hippocampus, new neurons are continually generated and incorporated into the circuitry throughout the lives of all mammals, including humans [1]-[11]. This form of structural and functional plasticity in the adult brain is believed to play an important role in hippocampal-dependent learning, memory and emotion [12]-[19]. Numerous studies have suggested that adult neurogenesis is regulated by a variety of physiological (e.g. aging, exercise, hormones or enriched environment), pathological (e.g. ischemia, injury, seizures or Alzheimer's disease) and pharmacological factors (e.g. antidepressants or pentobarbital) [for review see [9], [20], [21][. The mechanisms underlying the regulation of adult neurogenesis are still poorly understood.

The development of genetic tools that specifically label newborn dentate granule cells in adult mice is likely to facilitate the study of adult neurogenesis. For example, transgenic mice expressing GFP under the control of the nestin promoter have proved to be a valuable tool for studying neuronal progenitor cells [22]-[25]. More recently, transgenic mice expressing GFP in newborn dentate granule cells under the control of the proopiomelanocortin (POMC) or the doublecortin (DCX) promoter have been characterized [26]-[29]. These mice have greatly facilitated the study of the development and regulation of adult neurogenesis in the dentate gyrus [30]-[33].

Here we report a unique line of transgenic mice that selectively express GFP in newborn dentate granule cells in the hippocampus. Glutamate decarboxylase (GAD) is the rate-limiting enzyme that catalyzes the GABA synthesis from the decarboxylation of glutamate. In mammals GAD exists in two isoforms, GAD67 and GAD65. In attempting to label GABAergic neurons with GFP using a GAD67-GFP BAC transgenic approach, we discovered that in the hippocampus, our GAD67-GFP BAC transgene selectively labels cells in the subgranular zone of the dentate gyrus. Using BrdU staining, a new neuron marker, and morphological analysis we demonstrate that these GFP^+^ dentate granule cells are newborn neurons. These newly generated dentate granule cells are brightly labeled with GFP in their entirety including their cell bodies, full dendritic structures, and mossy fibers and their terminals, greatly assisting the study of the development and regulation of adult neurogenesis in the dentate gyrus. Thus the GAD67-GFP BAC transgenic mice could serve as a valuable genetic tool for studying various processes of adult neurogenesis in the dentate gyrus.

## Results

### The GAD67-GFP transgene labels the subgranular zone of dentate gyrus

**Figure 1:**
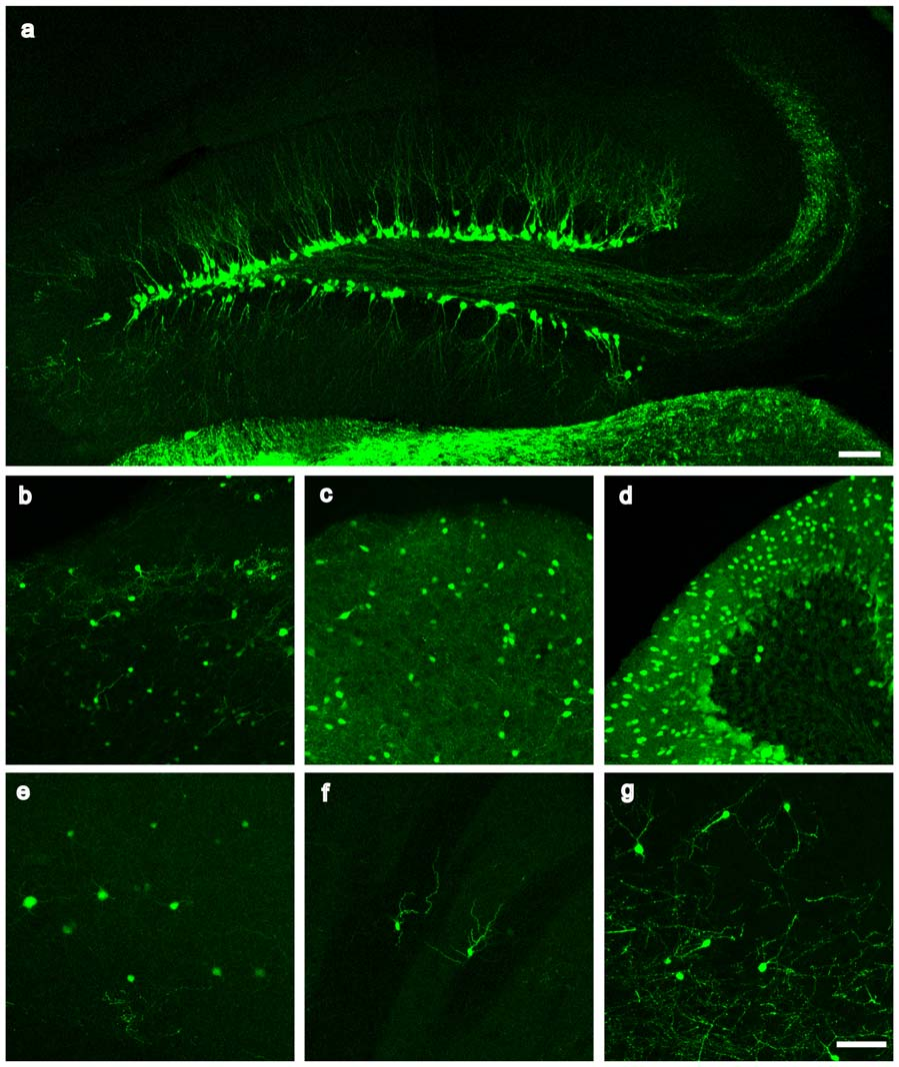
The GAD67-GFP transgene labels the subgranular zone of the dentate gyrus. **a,** GFP expression in the subgranular zone of the dentate gyrus of the hippocampus of a 3-month-old mouse. GAD67-GFP transgene labels the cells on the border of the dentate granule cell layer and hilus. The axons of the GFP-expressing dentate granule cells join the mossy fiber path and project to the stratum lucidum, while their dendrites reach the outer edge of the molecular layer. **b-g,** GFP expression pattern in various regions of 3-month-old mice. Z stack confocal images show GFP expression in olfactory bulb (**b**), superior colliculus (**c**), cerebellum (**d**), cortex (**e**), striatum (**f**) and thalamus (**g**). Scale bar, 100 µm.

In order to assist the study of the structure and function of GABAergic neurons, we generated BAC transgenic mice expressing EGFP under the control of GAD67 regulatory elements. We chose a BAC clone that contains the whole GAD67 gene plus 120 kb of 5′ upstream sequences and 46 kb of 3′ downstream sequences. We strategically replaced the initiating ATG codon of the GAD67 gene with the EGFP transgene, thus avoiding overexpression of GAD67 in the transgenic mice. Two lines of transgenic mice independently generated with this modified BAC show identical patterns of GFP expression in various regions of the brain. Confocal images without antibody enhancement show that GFP is strongly expressed in the Purkinje cells of the cerebellum, periglomerular neurons of the olfactory bulb, and interneurons of the superior colliculus and brainstem (Figure 1b-d and Figure S1). Sparse labeling is also seen in the thalamus, hypothalamus, cortex and striatum (Figure 1e-g and Figure S1). GFP fluorescence is clearly visible in the entirety of the labeled neurons, including cell bodies, axon terminals, and dendrites, thus facilitating the study of the morphology and structural plasticity of these neurons.

To our surprise, the expression of GFP in the hippocampus is restricted to the subgranular zone (SGZ) of the dentate gyrus (Figure 1a). Interneurons in the hippocampus, including those in the hilus, are excluded from GFP labeling. Two independently generated lines show an identical pattern of expression of hippocampal expression, suggesting that this is not due to a positional effect of transgene integration. Most of the GFP^+^ cells show typical dentate granule cell morphology and are located at the border of the dentate granule cell layer and the hilus. The apical dendrites of these neurons reach the outer edge of the molecular layer, and their axons join the mossy fiber path and extend to the CA3 region (Figure 1a).

**Figure 2:**
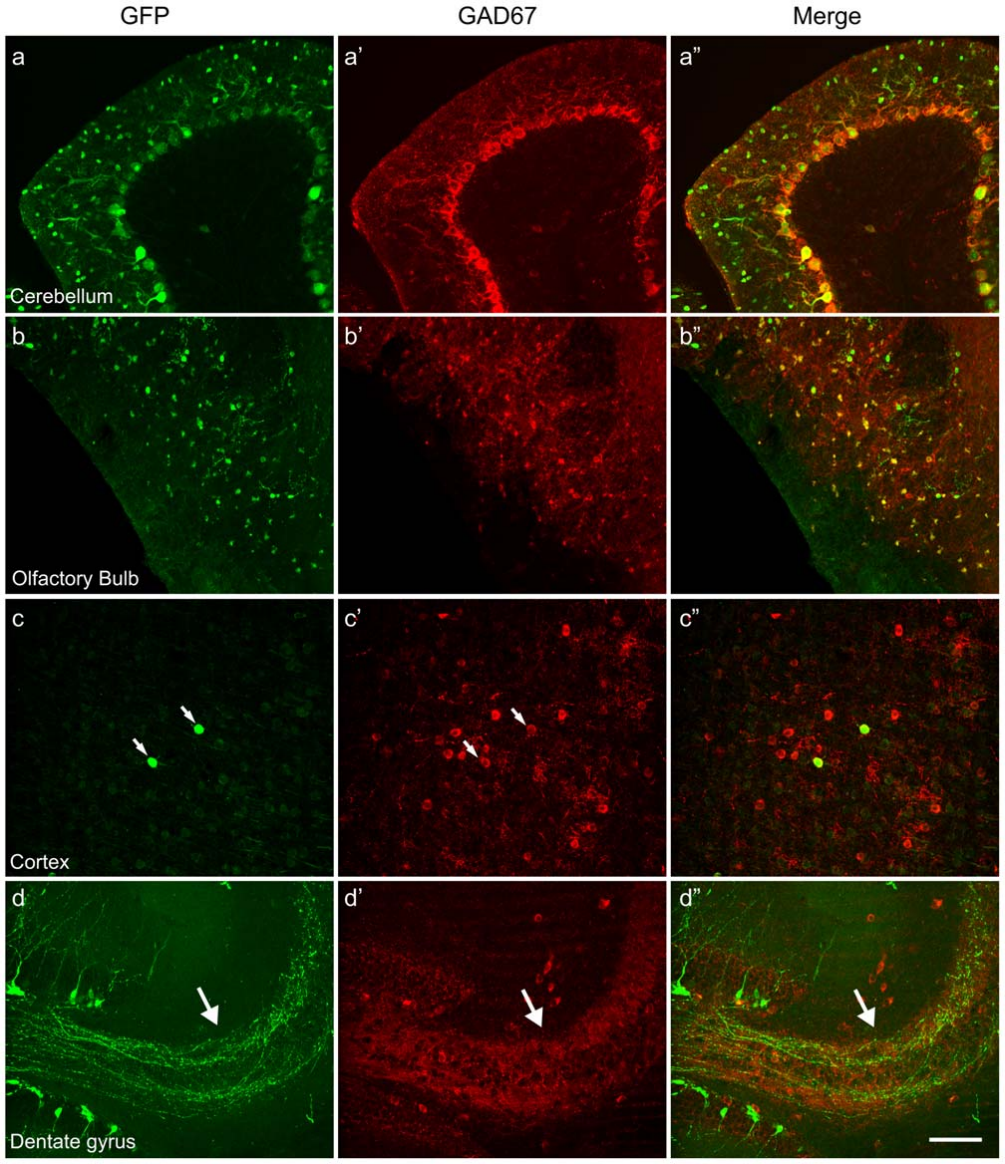
GFP labeled neurons in GAD67-GFP BAC transgenic mice are GABAergic neurons. Brain slices from 3-month-old mice were stained with a specific GAD67 antibody. Z stack confocal images show the colocalization of GFP fluorescence and GAD67 staining in cerebellar purkinje cells (a-a″), olfactory bulb periglomerular neurons (b-b″), and cortical interneurons (c-c″). In dentate gyrus, GFP expressing cells did not show strong GAD67 staining. However, the mossy fiber path shows a strong GAD67 signal (d-d″, arrows). Scale bar, 100 µm.

To determine whether the GFP labeled neurons in the GAD67-GFP BAC transgenic mice are GABAergic neurons, we stained brain slices with an anti-GAD67 specific antibody. Immunostaining showed a high degree of colocalization of GAD67 positive cells and GFP positive cells in the cerebellum, olfactory bulb, superior colliculus and midbrain (Figure 2a-b″ and data not shown), indicating that GFP is expressed in most GABAergic neurons in these regions of the brain. Although very few neurons are labeled with GFP in the cortex, thalamus, and striatum, they are all GAD67 positive (Figure 2c-c″ and data not shown). The bright and sparse labeling of GABAergic neurons in these regions of the brain could benefit structural and functional studies of these neurons.

Previous studies have shown that GAD67 and GABA are transiently expressed in dentate granule cells during development [34]-[36]. Our GAD67 immunostaining in the dentate gyrus did not show strong GAD67 immunoreactivity in the cell bodies of dentate granule cells. However, mossy fibers from the GFP^+^ dentate granule cells were clearly GAD67 positive (Figure 2d-d″). This may be due to the relatively low levels of GAD67 expression in dentate granule cells and is consistent with previous studies showing that GAD67 and GABA immunoreactivities are predominantly found in mossy fibers [35].

### GFP^+^ granule cells in GAD67-GFP BAC transgenic mice are newborn neurons

**Figure 3:**
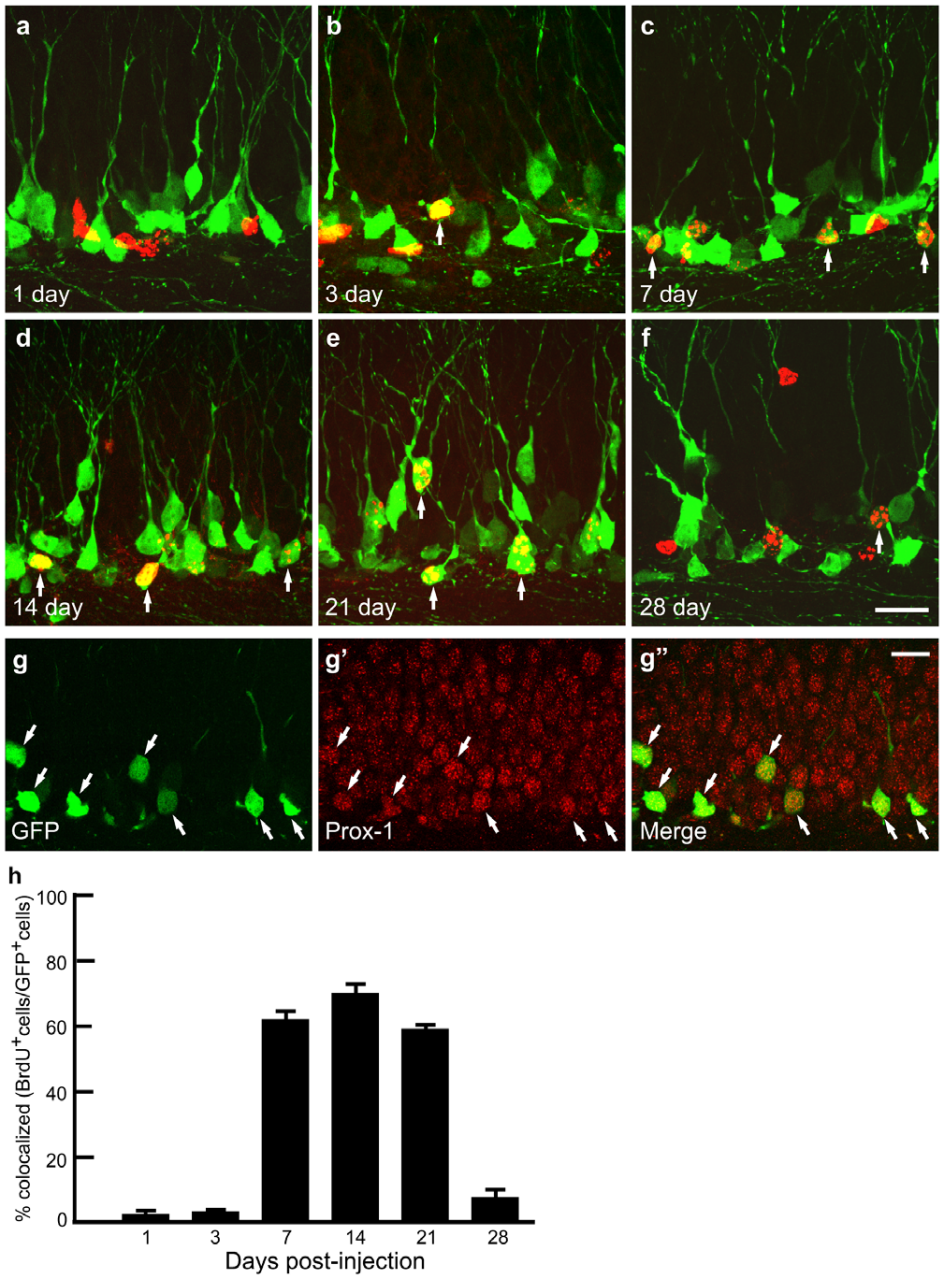
GFP+ granule cells are newborn neurons. **a-f,** BrdU-labeled nuclei (red) and GFP expressing cells (green) in the dentate gyrus at various stages after BrdU injection. Arrows show colocalization of BrdU-labeled nuclei and GFP expressing cells. **g,** All GFP+ cells express Prox-1, a dentate granule cell marker. **h,** Percentage of BrdU positive cells that are also GFP positive. Percentages of co-labeling are 1.3±0.3% at 1 day, 2.1±0.3% at 3 days, 62.5±3.2% at 1 week, 70.8±3.1% at 2 weeks, 58.7±1.3% at 3 weeks and 7.4±1.9% at 4 weeks (n = 4 mice each time point, mean±SEM). Scale bars, 20 µm.

The localization of GFP^+^ cells on the border of the dentate granule layer and hilus suggests that these are newly generated dentate granule cells. To test this, we first performed BrdU labeling in the GAD67-GFP BAC transgenic mice. One and three days after BrdU injection, a period of time that BrdU is incorporated into the chromosomes of proliferating neural progenitors, there was very little colocalization of BrdU-labeled cells with GFP positive cells (1.3% and 2.1% colocalization at 1 and 3 days, respectively; Figure 3a,b,h). One week after the injection, during which newborn neurons are being generated, the majority of the BrdU-labeled cells colocalized with GFP expressing cells (62.5%; Figure 3c,h). This high degree of colocalization persists 2 and 3 weeks after BrdU injection (70.1% and 58.7% for 2 and 3 weeks, respectively; Figure 3d,e,h). The number of BrdU and GFP colabeled cells dropped dramatically 28 days after injection (7.4%; Figure 3f,h), by which time most of the newborn neurons have become mature neurons [37], [38]. In addition, all GFP^+^ cells were positive for Prox-1, a dentate granule cell marker (Figure 3g-g″). Together, these data suggest that GFP^+^ cells are newly generated dentate granule cells and that GFP expression begins within the first week of cell division and disappears as newborn neurons mature, about 4 weeks postmitotic [37], [38].

**Figure 4:**
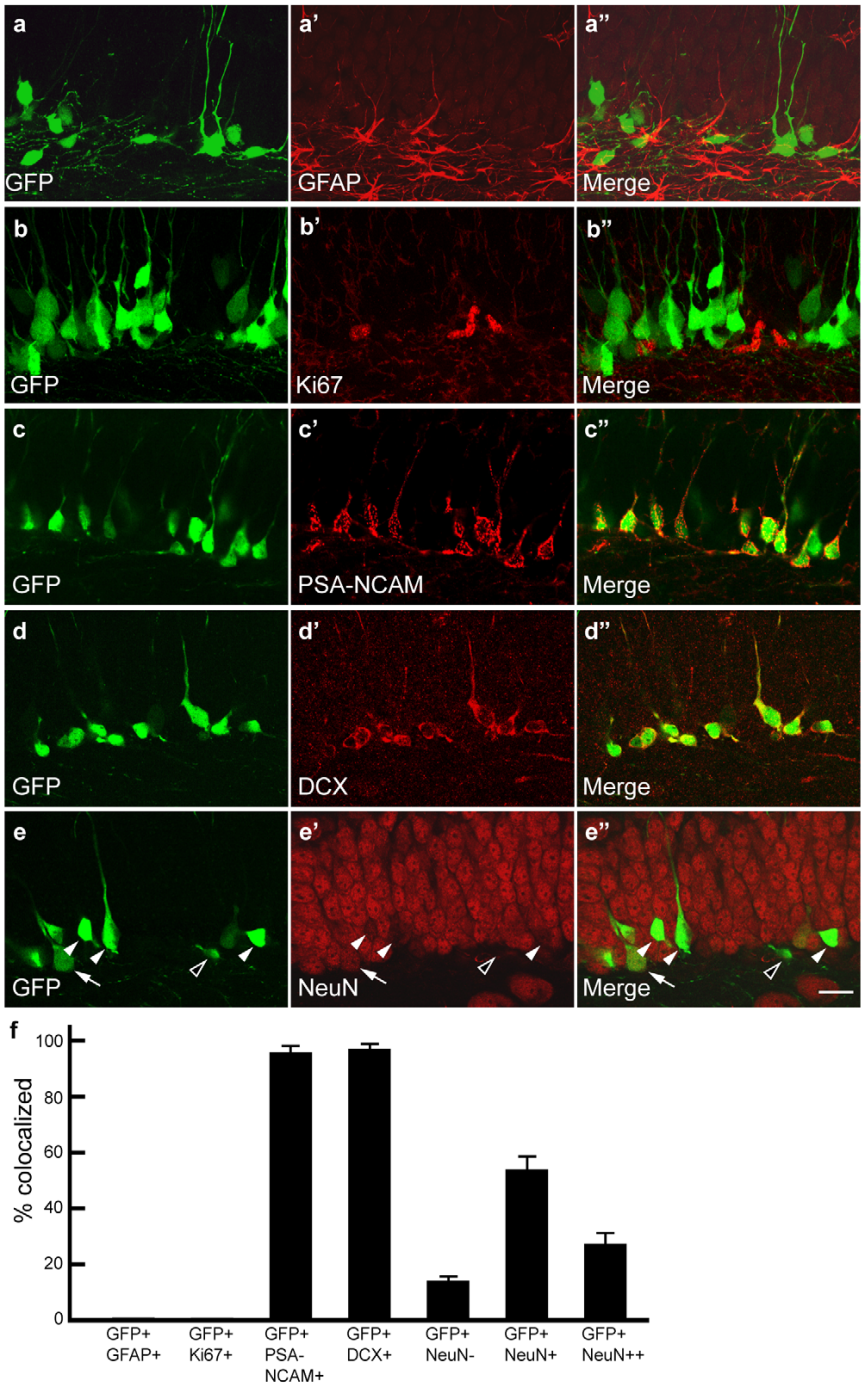
GFP+ granule cells express newborn neuron markers. **a-b″**, Confocal images show GFP^+^ cells do not colocalize with glial cell and neuronal stem cell marker GFAP (**a-a″**) or proliferating cell marker Ki67 (**b-b″**). **c-d″,** GFP^+^ cells precisely colocalize with newborn neuron makers PSA-NCAM (**c-c″**) and DCX (**d-d″**). **e-e″,** Many GFP^+^ cells are weakly positive for neuronal marker NeuN (closed arrowheads) while others are either negative (open arrowhead) or strongly positive (arrow). **f,** Quantification of colocalization of GFP^+^ cells with various cellular markers (mean±SEM). Images are single optical sections. Scale bar, 20 µm.

To further characterize the GFP^+^ dentate granule cells, we performed immunostaining for various cellular markers of different neuronal developmental stages. We found that GFP^+^ cells expressed neither GFAP (Figure 4a-a″,f), a marker for glial cells and neuronal stem cells, nor Ki67, a marker for proliferating cells, indicating that these GFP^+^ cells are postmitotic (Figure 4b-b″,f). However, the vast majority of GFP^+^ cells expressed both doublecortin (DCX; 96.9%) and PSA-NCAM (96.3%), two well characterized newborn neuron markers (Figure 4c-c″,d-d″,f). In addition, staining for NeuN, a neuronal marker highly expressed in mature neurons, revealed that most GFP^+^ cells were either weakly positive (53.4%) or negative (13.8%), suggesting that they are in the process of transitioning to become mature neurons (Figure 4e-e″,f). Together with the BrdU staining results, these data indicate that GFP^+^ cells in the dentate gyrus of GAD67-GFP mice are postmitotic newborn neurons that appear to be at advanced developmental stages (late stage 4 to stage 5) [6], [7], [9], [20].

The subventricular zone (SVZ), rostral migratory stream (RMS) and the olfactory bulb are the brain regions which also involve active adult neurogenesis (for review, see 39]. To further investigate whether the GFP^+^ periglomerular neurons and granular neurons labeled in this mouse line are new born neurons, we did the doublecortin staining and BrdU labeling. Immunostaining with doublecortin, a newborn neuron maker, shows the specific immunofluorescence in dentate gyrus of hippocampus and the SVZ-RMS-olfactory bulb pathway. Doublecortin precisely co-localized with GFP^+^ cells in dentate gyrus (Figure 4.d-d’’). However, no co-localization of doublecortin and GFP^+^ cells in the SVZ-RMS-olfactory bulb pathway was found [Figure S3a-a’’ and data not shown). Consistent with doublecortin staining, BrdU labeled new born neurons in olfactory bulb and the SVZ-RMS showed no co-localization with GFP^+^ cells either [Figure 3b-b’’), while they co-localize well in dentate gyrus (Figure 3c-e), demonstrating that the GFP^+^ periglomerular neurons and granular neurons in this line are not newborn neurons.

**Figure 5:**
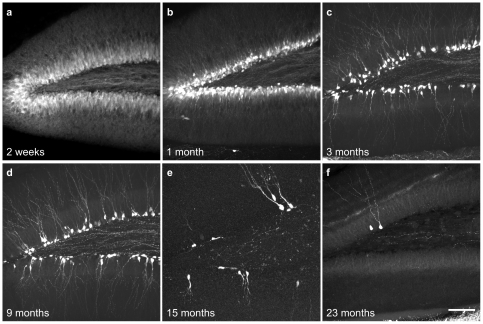
The developmental changes of GFP+ newborn dentate granule cells. Z stack confocal images show the developmental changes of GFP+ newborn neuron numbers at 2 weeks, 1 month, 3 months, 9 months, 15 months and 23 months. Scale bar is 100 µm.

In rodents, most dentate granule cells are generated within the first month of birth [40], [41]. After this period, the rate of neurogenesis decreases dramatically with age. To examine whether GFP^+^ dentate granule cells also show similar developmental changes, we imaged GFP^+^ cells in GAD67-GFP BAC transgenic mice at various developmental stages. Two weeks after birth, GFP-expressing neurons cover almost the entire granule cell layer, indicating massive neurogenesis at this stage (Figure 5a and Figure S2a). At 1 month of age, the number of GFP-expressing new neurons is significantly reduced and they are restricted to the inner third of the dentate granule cell layer (Figure 5b and Figure S2b). In 3 to 9-month-old mice, the number of the GFP^+^ cells decreases dramatically and they only line the inner granule cell layer (Figure 5c,d and Figure S2c,d). The number of GFP^+^ dentate granule cells further decreases with age, and by 15 months and older, only a few GFP^+^ cells are visible (Figure 5e,f). However, the number of GFP^+^ cells in the other brain regions remains relatively constant throughout the time period investigated (Fig. S2a-d). This developmental profile is consistent with the postnatal development of dentate granule cells [41] as well as the age-related reduction of neurogenesis in the adult dentate gyrus [26], [42] further supporting our conclusion that the GAD67-GFP transgene specifically labels newly generated dentate granule cells. Consistent with the previous report [43], GFP^+^ newborn neuron quantification shows no significant difference between the two hemispheres, while the dorsal blade has more newborn neurons than the ventral blade (Table S1).

### GFP^+^ newborn neurons show immature morphology

**Figure 6:**
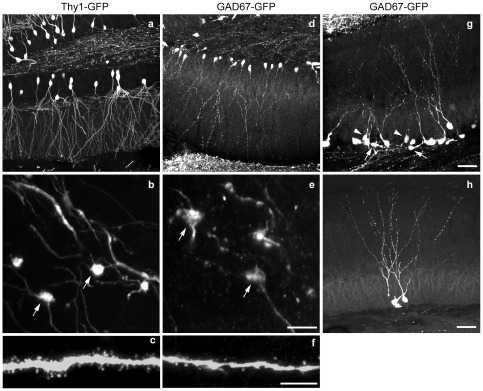
GFP+ newborn neurons show immature morphology. **a-c,** GFP-expressing mature dentate granule cells from 9 month-old Thy1-GFP mice show short primary dendrites, multiple dendritic branches (**a**), large presynptic boutons in the CA3 region (**b**), and spiny dendrites (**c**). **d-f,** GFP-expressing newborn dentate granule cells from 9 month-old GAD67-GFP mice show long primary dendrites, fewer dendritic branches, and numerous dendritic varicosities (**d**). High magnification images also reveal the mossy fiber giant boutons in the CA3 region (**e**), dendritic beading and fewer spines (**f**). **g,** The heterogeneity in morphology of GFP-expressing newborn neurons in 3 month-old GAD67-GFP mice. Arrows indicate the new neurons with irregular shaped somas and short processes that are parallel to the dentate granule cell layer. Arrowheads indicate the newborn neurons with small somas and short dendrites terminating in the inner molecular layer; the majority of the newborn neurons have long primary dendrites and more branches projecting to the outer edge of the molecular layer. h, GFP expressing newborn neurons in a 23-month-old mouse. Scale bar is 50 µm in **g** for **a**, **d**, and **g**; 10 µm in **e** for **b** and **e**; 5 µm in **f** for **c** and **f**; and 50 µm in **h**.

To further characterize the developmental stages of GFP^+^ newborn neurons, we analyzed their morphological features and compared them with mature dentate granule cells from Thy1-GFP-M mice, which selectively express GFP in subsets of mature dentate granule cell [44]. To better visualize the morphology of single neurons and their processes, we used 7-9 month old mice, which have fewer GFP expressing cells. Confocal imaging clearly shows that most GFP^+^ dentate granule cells from GAD67-GFP mice line the border between the dentate granule cells and the hilus, with their dendrites spanning the entire molecular layer and reaching its outer edge (Figure 6d). Compared to mature dentate granule cells in Thy1-GFP-M mice, dendrites of newborn granule cells show immature dendritic morphology including longer primary dendrites (51.39±4.70 µm vs. 12.11±2.29 µm, *p*<0.001, Figure S4D), fewer branches (6.2±0.35 µm vs. 9.25±0.60 µm, *p*<0.001, Figure S4C), shorter total dendritic length (595.51±37.02 µm vs. 1131.36±57.02, *p*<0.001, Figure S4B) and numerous dendritic varicosities (Figure 6a,d). GFP^+^ granule cells in the GAD67-GFP mice also show morphological heterogeneity. Some have irregularly shaped somas with very short dendrites parallel to the granule cell layer (Figure 6g, arrows), some have small somas and short dendrites terminating in the inner molecular layer (Figure 6g arrowheads), but the majority have large somas with long primary dendrites and several branches projecting to the outer edge of the molecular layer (Figure 6d,g,h).

High magnification images revealed that the dendrites of GFP^+^ newborn granule cells show dendritic beading and fewer spines, in contrast to the spiny dendrites of mature granule cells (Figure 6c,f). The axons of GFP^+^ newborn granule cells extend to the farthest end of the stratum lucidem (Figure 1h), and mossy fiber giant boutons are readily visible in the CA3 region (Figure 6e, arrows), suggesting that they form synaptic connections with CA3 pyramidal neurons. Unlike the immature morphology of their dendrites, the mossy fiber giant boutons of the newborn neurons are similar to those of mature dentate granule cells (Figure 6b,e), providing direct evidence to support the notion that dendrites of developing dentate granule cells mature after their axons reach the CA3 region [45]. Together, these morphological data provide additional evidence that GFP^+^ cells are newborn neurons and that many of them have reached advanced stages of development [7], [20], [46]-[48]. The labeling of newborn neurons in advanced stages with GFP will likely facilitate the study of structural changes of these neurons under physiological as well as pathological conditions.

### GFP^+^ newborn neurons show immature electrophysiological properties

**Figure 7:**
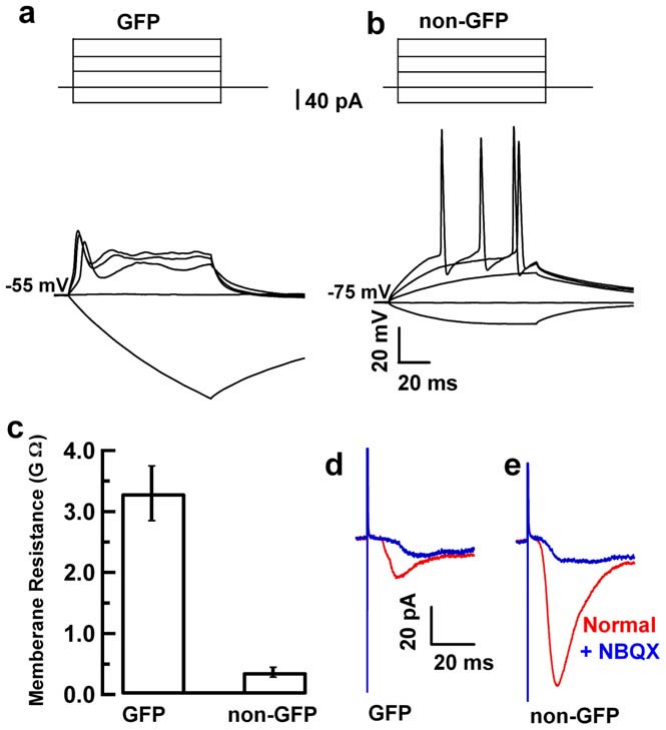
GFP+ newborn neurons show electrophysiological properties of immature neurons. **a**, **b**, Current injection induced small, non-typical action potentials in GFP^+^ newborn neurons (**a**) and trains of typical action potentials in the non-GFP^+^ mature granule cells (**b**). **c**, GFP^+^ cells show larger membrane resistance (3.3±0.45 GΩ, n = 13), while non-GFP mature granule neurons show lower membrane resistance (0.37±0.08 GΩ, n = 10). **d**, **e**, Stimulation of the perforant pathway evoked large response of EPSCs in non-GFP neurons (**e**), but very small EPSCs, or no EPSCs in GFP^+^ neurons (**d**). Recordings with and without NBQX are indicated in blue and red curves.

Previous studies have shown that adult-born neurons in the subgranular zone of the dentate gyrus have slower membrane time constants, higher input resistance and more depolarized resting membrane potentials [26], [49]. To determine the functional properties of the GFP^+^ newborn neurons in the GAD67-GFP line, we did whole cell recordings using acute hippocampal slices, and compared these recordings to neighboring non-GFP^+^ granule cells from adult mice. Unlike mature granule cells, which generate trains of action potential during current injection, GFP^+^ neurons only generate a few non-typical action potentials. The current threshold to evoke an action potential is lower in GFP^+^ neurons than in non- GFP mature neurons (Figure 7a,b). Additionally, these GFP^+^ cells have larger membrane resistance values (3.3±0.45 GΩ, n = 13) compared with non-GFP mature granule neurons (0.37±0.08 GΩ, n = 10) (Figure 7c). Stimulation of the perforant pathway evoked large responses of EPSCs in non-GFP neurons, but only very small EPSCs in GFP^+^ neurons, and sometimes, no EPSCs could be evoked in some GFP^+^ neurons (if the cell morphology is very simple) (Figure 7d,e). Our electrophysiological results are consistent with previous publications [26], [49], [50], which further validated that GFP^+^ neurons in SGZ of GAD67-GFP transgenic mice are newborn neurons.

**Figure 8:**
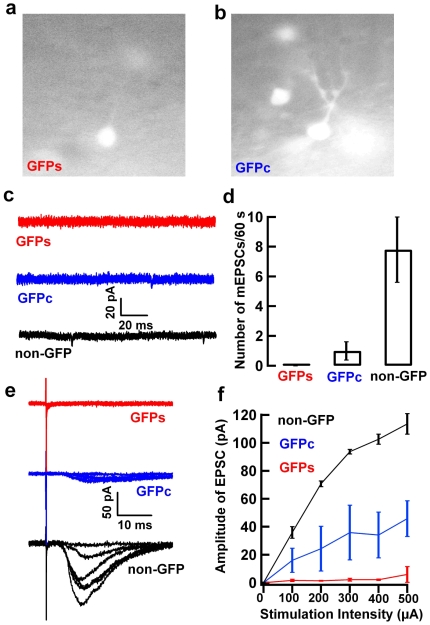
GFP+ newborn neurons are functionally distinct at different developmental stages. **a**, **b**, Representative pictures of GFP^+^ neurons at early stage with simple branches (GFPs) (**a**) and late stage with complex branches (GFPc) (**b**). Pictures were taken from live slices by a CCD camera coupled to the microscope. **c**, **d**, Representative traces (**c**) and statistical results (**d**) of miniature EPSCs. No miniature EPSCs were recorded from GFPs neurons (red, n = 5), and few miniature EPSCs were recorded from GFPc neurons (blue, n = 5) as compared with non-GFP mature granule cells (black, n = 6). **e**, **f**, Typical example (**e**) and statistical analysis (**f**) of evoked EPSCs. While stimulation of perforant pathway evoked robust response of EPSCs in non-GFP mature granule cells (black, n = 6), very small EPSCs were evoked in GFPc neurons (blue, n = 5), and no response could be observed in GFPs neurons (red, n = 6).

We further characterized synaptic integration of GFP^+^ newborn neurons at differential developmental stages. We examined the miniature EPSCs and the evoked EPSCs from GFP^+^ neurons at early stage with simple branches (GFPs) and late stage with complex branches (GFPc). Representative pictures of live GFPs and GFPc neurons with patch pipette are shown in figure 8a, b. Different from non-GFP mature granule neurons, no miniature EPSCs were recorded from GFPs neurons, and few miniature EPSCs were recorded from GFPc neurons (Figure 8c,d). While stimulation of the perforant pathway evoked robust response of EPSCs in non-GFP neurons, very small EPSCs were evoked in GFPc neurons, and no response could be observed in GFPs neurons (Figure 8e,f). Consistent with our morphological investigation (Figure 6), these results further demonstrate the heterogeneity and the immaturity of the GFP^+^ newborn neurons.

## Discussion

### Transgenic markers for newborn dentate granule cells

An important technical development in the study of adult neurogenesis is the generation of transgenic mice that specifically express fluorescent proteins in newborn dentate granule cells. Transgenic mice expressing GFP under the control of the nestin promoter have proved to be useful tools to identify progenitor cells in the adult hippocampus. In these mice, GFP selectively labels type-1 and type-2 progenitor cells, but not postmitotic neurons [22]-[24]. More recently, transgenic mice expressing GFP under the control of the POMC promoter have been shown to selectively and transiently label newborn dentate granule cells [26], [51]. In POMC-GFP mice, labeled cells have primary dendrites that branch and terminate within the inner molecular layer, and mossy fiber axons that terminate in the stratum lucidem without mature mossy fiber boutons. Colocalization with BrdU indicates that GFP-labeled granule cells are ∼2 weeks postmitotic. The labeling of newborn neurons with GFP in POMC-GFP mice has greatly facilitated the study of adult neurogenesis [30]-[33]. Most recently, transgenic mice expressing DsRed under the control of the DCX promoter have also been reported. Although neuronal processes are not well labeled in these mice they facilitate the identification of newborn neurons for functional characterization [27].

Our GAD67-GFP BAC transgenic mice provide another genetic tool for studying adult neurogenesis, and these mice have several unique features. First, unlike Nestin-GFP mice, our data suggests that only postmitotic newborn neurons, not proliferating progenitor cells, are labeled in GAD67-GFP mice. Second, unlike labeled cells in POMC-GFP mice, DCX-GFP mice and DCX-DsRed mice, many GFP^+^ cells in the GAD67-GFP mice show dendritic structures in the advanced stages of development, approaching maturation. They have multiple branches, reach the outer edge of the molecular layer, and some have a few spines. Third, the axons of GFP^+^ cells in the GAD67-GFP mice also show more mature features than those in POMC-GFP mice, including numerous giant mossy fiber boutons. Therefore, different from the POMC-GFP mice, which only labels the new-born neurons up to two weeks postmitotic, our GAD67-GFP transgene labels the whole developmental stage of newborn neurons. GFP expression begins within the first week of cell division and disappears as newborn neurons mature, about 4 weeks postmitotic. The labeling of more mature features of the dendrites and axons of newborn neurons may facilitate the study of their modulation and functional integration. Thus, the GAD67-GFP mice serve as a valuable genetic tool for studying adult neurogenesis, complementary to nestin-GFP, DCX-DsRed, DCX-GFP and POMC-GFP mice. Together with Thy1-GFP mice, in which mature dentate granule cells are specifically labeled, these groups of transgenic mice provide unique tools for studying dentate granule cells at different developmental stages.

### Transcriptional regulation of GAD67

The regulatory elements of the GAD67 gene have been intensively studied, and several transgenic reporter mice have been generated using various fragments of the GAD67 5′ genomic sequences or large BAC clones [52], [53]. However, all GAD67 transgenic reporter mice only label either a random or specific subset of GAD67 positive neurons, suggesting the existence of complex mechanisms for GAD67 expression. The BAC clone used in the current study contains 120 kb of 5′ upstream and 46 kb of 3′ downstream sequences, yet it did not recapitulate the endogenous expression pattern of the GAD67 gene. The selective labeling of newborn dentate granule cells in the GAD67-GFP BAC transgenic mice suggests that there might be specific regulatory elements controlling GAD67 expression in these newborn neurons. Because the endogenous GAD67 gene is expressed in newborn dentate granule cells during development (see below), the expression of GFP in newborn dentate granule cells of our GAD67-GFP mice could be due to the inclusion of positive regulatory elements in the BAC clone, and the high levels of GFP expression relative to the endogenous GAD67 gene expression in these cells may be attributed to multiple copies of the transgene in the mice or the relatively stable nature of the GFP protein. Alternatively, it could be due to the loss of negative regulatory elements in the BAC clone that would lead to enhanced GAD67-GFP transcription activity in newborn dentate granule cells. Interestingly, in GAD67-GFP knock-in mice, which contain all endogenous regulatory sequences, there is no GFP expression in newborn dentate granule cells [54, S. Zhao and G. Feng, unpublished observation]. Thus, it is likely that our GAD67 BAC clones lack some negative regulatory elements which leads to the enhanced expression of GFP in newborn dentate granule cells.

Dentate granule cells are traditionally considered to be glutamatergic neurons, and they form excitatory synapses on CA3 pyramidal cells and local inhibitory interneurons of the hilar and CA3 regions [55], [56]. However, several studies have shown that these glutamatergic dentate granule cells also express GAD67 and VGAT mRNA. The mRNA levels of GAD67 and VGAT in granule cells are developmentally and activity-dependently regulated [36], [57], [58]. GABA and GAD67 have been found in dentate granule cells and mossy fiber terminals, and GABA and glutamate colocalize within the same mossy fiber terminals [34], [35]. Studies have also shown the co-release of both glutamate and GABA in the dentate granule cells and neurons in the auditory brainstem [59], [60]. The GABAergic phenotype of the dentate granule cells shows developmental and activity-dependent regulation. During the first 3 postnatal weeks, mossy fiber stimulation provokes monosynaptic fast inhibitory transmission mediated by GABA, in addition to monosynaptic excitatory glutamatergic transmission, onto CA3 pyramidal cells. After this stage, mossy fiber GABAergic transmission abruptly disappears and GABAergic markers are undetectable [61], suggesting that GABA expression is transient during the development of dentate granule cells. The expression of GFP in dentate granule cells of our GAD67-GFP mice during postnatal development is consistent with the expression profile of endogenous GABA expression, providing supporting evidence of transcriptional activity of the GAD67 gene in developing dentate granule cells.

GABA is known to play an important role in the developing brain. In the dentate gyrus, in addition to glutamate, granule cells release GABA during early development. The exact function of the co-release of glutamate and GABA from dentate granule cells is still not well understood, but it has been proposed to provide neurons additional means of regulating synaptic development and plasticity [36], [61], [62]. For examples, before P10, GABA release produces excitatory postsynaptic potentials, but becomes inhibitory after that. Thus, release of GABA from young and mature granule cells has opposite effects on excitability of postsynaptic cells. The transient GABA phenotype of adult-born neurons likely serves a similar role in regulating synaptic development, maturation and plasticity.

## Materials and Methods

### Antibodies

Rabbit anti-GFP antibody (1∶5000 dilution) was a gift of Dr. Don Lo, Duke University, Durham, NC. Mouse anti-GAD67 (MAB5406, 1∶1000 dilution), mouse anti-PSA-NCAM (MAB5324, 1∶500 dilution), mouse anti-NeuN (MAB377, 1∶200 dilution), and guinea pig anti-DCX (AB2253, 1∶1000 dilution) antibodies were from Chemicon (Temecula, CA). Rabbit anti-GFAP antibody (ab7260, 1∶200 dilution) was from Abcam. Rat anti-BrdU antibody (monoclonal, Cat# OBT0030, 1∶200 dilution) was from Accurate Scientific (Westbury, NY). Mouse anti-Ki67 (Cat#556003, 1∶200 dilution) antibody was from BD Biosciences. Rabbit anti-Prox-1 (PRB-238C-200, 1∶5000) was from Covance (Austin, TX).

### Generation of GAD67-GFP BAC transgenic mice

A BAC clone containing the GAD67 gene plus 120 kb of 5′ upstream and 46 kb of 3′ downstream sequences (Clone-ID: RP23-118N13) was obtained from the BACPAC Resource Center (Oakland, CA). EGFP sequence with bovine growth hormone 3′ untranslated region and polyadenylation signals was placed in exon 2 using a RecA homologous recombination system [63] developed by the laboratory of Dr. Nathaniel Heintz (Rockefeller University). Transgenic mice were generated by the injection of modified BAC DNA constructs into fertilized oocytes, using standard pronuclear injection techniques [64]. Fertilized eggs were collected from the matings between C57BL/6J and CBA F1 hybrids. Genotypes were determined by PCR from mouse tail DNA samples. A forward primer from the mouse GAD67 gene (GAD67AF2, 5′-GTCTCACCAAAGTCCCTGTCC-3′) and a reverse primer from the GFP sequence (YFPR2, 5′-CGTCCTCCTTGAAGTCGATGCC-3′) were used for genotyping. PCR-positive animals were kept as founders to establish transgenic lines by mating to C57BL/6J mice. The mice have been deposited to The Jackson Laboratory (Stock number 007673). All research involving mice have been conducted according to the Institutional Animal Care and Use Committee guidelines at Duke University. All procedures were approved by the Institutional Animal Care and Use Committee at Duke University.

### Section preparation and imaging

Mice were anesthetized by the inhalation of isoflurane and were intracardially perfused with 30 ml Lactated Ringers solution, followed by 30 ml 4% paraformadehyde (PFA). Mouse brains were then post-fixed in 4% PFA overnight at 4°C. 50 µm sagittal sections were cut using a vibrotome. Rabbit anti-GFP antibody was used to enhance the GFP fluorescence in co-immunostaining with the other antibodies (Figure 2,3,4). Briefly, sections were blocked with blocking buffer (5% normal goat serum, 2% BSA, 0.2% triton X-100 in PBS) for 1 hour at room temperature, then incubated with rabbit anti-GFP antibody overnight at 4°C. Following incubation with the first antibody, sections were washed with PBS 3 times every 20 minutes, followed by incubation with FITC-conjugated goat anti-rabbit secondary antibody for 2-4 hours at room temperature, and then washed with PBS. Sections were transferred onto slides, dried, mounted with 0.1% paraphenylinediamine in 90% glycerol/PBS (PPD), and imaged with a confocal microscope (Nikon). For co-staining experiments, sections were incubated either with mouse anti-NeuN, mouse anti-PSA-NCAM or rabbit anti-GFAP antibody, then with Cy3-conjugated goat anti-mouse secondary antibody. GAD67 staining was done as previously described [35]. Briefly, 50 µm vibrotome-cut slices were transferred to 12 well plate, rinsed in 0.1M Tris (pH7.6) three times for 5 minutes each with gentle shaking, blocked in Tris-BSA for 15 min (0.1M Tris, pH7.6, 0.005% BSA), then blocked with 10% normal goat serum in Tris-BSA for one hour. Slices were then incubated with mouse anti-GAD67 antibody (1∶1,000 dilution) in Tris-BSA buffer at 4°C overnight with gentle shaking and washed in 0.1M Tris three times for 10 minutes each. For second antibody incubation, slices were incubated with goat anti-mouse Cy3 second antibody (1∶1,000) at room temperature for two hours, followed by the same wash as above, then mounted on microscope slides (Fisher Scientific) with PPD and imaged with fluorescent microscope.

### BrdU labeling and Immunostaining

2-3 month old mice were intraperitoneally injected with 50 mg/kg BrdU (Sigma) four times within 8 hours. Animals were sacrificed 1, 3, 7, 14, 21 and 28 days after BrdU injection, and brain sections were prepared as above. To achieve a better imaging result, co-immunostaining was done sequentially. First, sections were stained with rabbit anti-GFP antibody as described above, then fixed with 4% PFA for 10 minutes to minimize the signal loss caused by the later steps, then rinsed with PBS 3 times for 5 minutes each, and stained with rat anti-BrdU antibody as previously described [65]. Briefly, sections were incubated in 50% formamide/2xSSC at 65°C for 2 hours, rinsed in 2xSSC for 5 minutes, incubated in 2N HCl at 37°C for 30 minutes, then rinsed in 0.1 M boric acid (pH 8.5) for 10 minutes. Sections were washed in TBS (0.15 M NaCl and 0.1 M Tris-HCl, pH 7.5) for 30 minutes, blocked with 3% heat inactivated normal rabbit serum/0.1% Triton X-100 in TBS (TBS-HINRS) for 30 minutes, followed by incubation with rat anti-BrdU antibody in TBS-HINRS for 48 hours at 4°C. After incubation with the first antibody, sections were washed in TBS 3 times for 10 minutes each, incubated with biotinylated rabbit anti rat IgG (Vector labs, 1∶100 dilution) for 2 hours, then incubated with Texas Red Streptavidin in TBS + 0.1% Triton X-100 for 2 hours. After the TBS wash, sections were transferred onto slides, mounted and imaged as described above.

### Electrophysiology

Mice (2∼3 months old) housed under standard condition were processed for slice preparation and electrophysiological experiments were performed as previously described [49]. Recordings were obtained at 32°C. GFP positive neurons in subgranule or granule cell layer were identified by green fluorescence. Microelectrodes (4–5MΩ) were filled with the following pipette internal solution: 120 mM potassium gluconate, 15 mM KCl, 4 mM MgCl_2_, 0.1 mM EGTA, 10.0 mM HEPES, 4 mM Mg ATP, 0.3 mM Na_3_GTP, 7 mM phosphocreatine (pH 7.4, 300 mOsm). Data were collected using an Axon 700B amplifier and acquired with a DigiData 1440 (Axon Instruments) at 10 kHz. Series resistances were continuously monitored, series resistance ranged from 10–20 MΩ was uncompensated, only those neurons with changes of series resistance less than 15% during experiments were analyzed. To examine the evoked synaptic transmission, a concentric bipolar electrode (FHC Inc) was used to stimulate the perforant pathway input to dentate gyrus. Stimulation was delivered by Axon DigiData through isolator (Iso-flex, AMPI). For measurement of EPSCs, stimulation intensity (400 µA, 100 µs duration)) was maintained and five consecutive traces were averaged for presentation. NBQX (10 µM) was used to block non-NMDA mediated EPSCs. Input-output curves were examined by increasing the stimulation intensity with 100 µA intervals starting from 0 µA.

## Supporting Information

Table S1 Distribution of GFP^+^ newborn neurons in the dentate gyrus. Serial sections from three mice aged at two to three months were cut. Slices were imaged on a Zeiss Axioskop 2 microscope, then GFP^+^ newborn neurons from every sixth section were counted using Image-Pro Plus software. The resulting number was then multiplied by 6 and statistics was done with Student's t-test. The dorsal blade shows significantly more GFP^+^ newborn neurons than the ventral blade (P = 0.00012). No significant differences were found in GFP^+^ newborn neurons between the two hemispheres (P = 0.975). ** Indicates a significant difference.
(0.02 MB DOC)

Figure S1 Serial sagittal brain images of GAD67-GFP mouse. A-K. Serial sagittal brain images of eleven sections spaced 300 µm apart. Sections are from a male mouse aged 2.5 months with a C57/BL6 genetic background. Whole brain montages were generated with a Zeiss motorized stage.
(0.43 MB DOC)

Figure S2 The developmental changes of GFP^+^ neurons. A-D. Whole mouse brain images from GAD67-GFP mice at the age of two weeks (A), one month (B), three months (C) and nine months (D). 50 µm sections were from a male mouse with a C57/BL6 genetic background. Whole brain montages were generated with a Zeiss motorized stage.
(0.50 MB DOC)

Figure S3 GFP^+^ cells in olfactory bulb are not newborn neurons. a-b'. Brain sections from a 2-3 month old mouse were stained with GFP (a, b), doublecortin (DCX) (a') and BrdU (b'). a' and b' show the merged images. Images were taken with Olympus FV-1000 confocal microscope using a 20× objective. Scale bar is 100 µm. Neither of doublecortin staining (a') or BrdU labeling (b') colocalized with GFP^+^ cells in the olfactory bulb.
(3.55 MB DOC)

Figure S4 Dendritic quantification of dentate granule neurons from Thy1-GFP mice and GAD67-GFP mice. Thy1-GFP-M line and GAD67-GFP line mice around 7-month-old were paired. For a better quantification, anti-GFP antibody was used to enhance the signal as described above. GFP fluorescence was imaged with an Olympus BX61WI confocal microscope, and dendritic branch numbers and dendritic length were quantified with Nurolucida 9 software (MBF Bioscience). A. Representative examples of dentate granule neurons in Thy1-GFP-M and GAD67-GFP transgenic mice. Scale bar is 20 µm. B-D. Quantitative analysis of total dendritic length (B), branch number (C), and primary dendritic length (D) of dentate granule neurons in Thy1-GFP-M and GAD67-GFP transgenic mice. GAD67-GFP labeled newborn neurons show significantly fewer dendritic branches (6.2±0.35 µm vs. 9.25±0.60 µm, p<0.001) and shorter total dendritic length (595.51±37.02 µm vs. 1131.36±57.02, p<0.001) than Thy1-GFP labeled mature dentate granule neurons. However, they show longer primary dendrites than Thy1-GFP positive mature granule cells (51.39±4.70 µm vs. 12.11±2.29 µm, p<0.001). Data represent Mean±SEM; n = 20 for each group (** p<0.001, student t test).
(0.19 MB DOC)
