# Supplementary material for: Fluorescent Labeling of Newborn Dentate Granule Cells in GAD67-GFP Transgenic Mice: A Genetic Tool for the Study of Adult Neurogenesis
Source: PLoS One. 2010 Sep 2;5(9):e12506. doi: 10.1371/journal.pone.0012506 (PMC2932690; doi:10.1371/journal.pone.0012506)
Supplement: Table S1 — Distribution of GFP+ newborn neurons in the dentate gyrus. Serial sections from three mice aged at two to three months were cut. Slices were imaged on a Zeiss Axioskop 2 microscope, then GFP+ newborn neurons from every sixth section were counted using Image-Pro Plus software. The resulting number was then multiplied by 6 and statistics was done with Student's t-test. The dorsal blade shows significantly more GFP+ newborn neurons than the ventral blade (P = 0.00012). No significant differences were found in GFP+ newborn neurons between the two hemispheres (P = 0.975). ** Indicates a significant difference. (0.02 MB DOC) [file pone.0012506.s001.doc]

**Table S1. Distribution of GFP^+^ Newborn Neurons in the Dentate Gyrus**

Area Mean ±SEM Mean difference *P* Value

Dorsal blade 5128 ± 266.1 2217 0.00012**

Ventral blade 2977 ± 280.9

Left GCL 8076 ± 1048.8 -36 0.9750

Right GCL 8112 ± 838.0
