# Supplementary figures and images for: Fluorescent Labeling of Newborn Dentate Granule Cells in GAD67-GFP Transgenic Mice: A Genetic Tool for the Study of Adult Neurogenesis
Source: PLoS One. 2010 Sep 2;5(9):e12506. doi: 10.1371/journal.pone.0012506 (PMC2932690; doi:10.1371/journal.pone.0012506)

**Figure S1. Serial sagittal brain images of GAD67-GFP mouse**


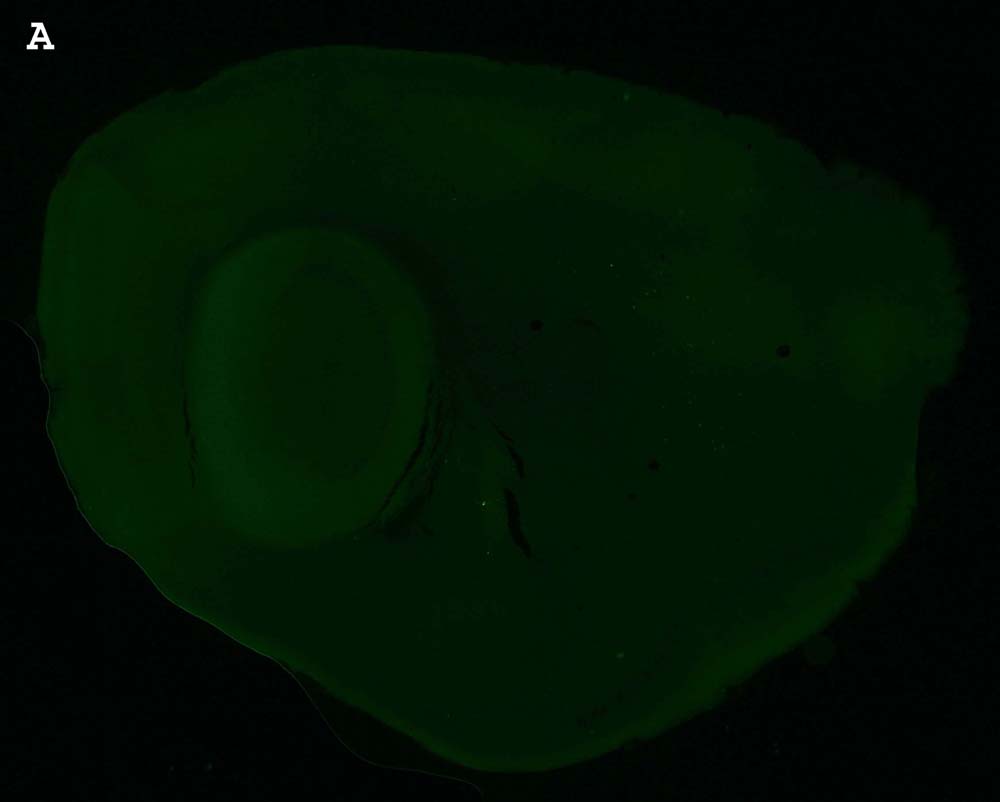


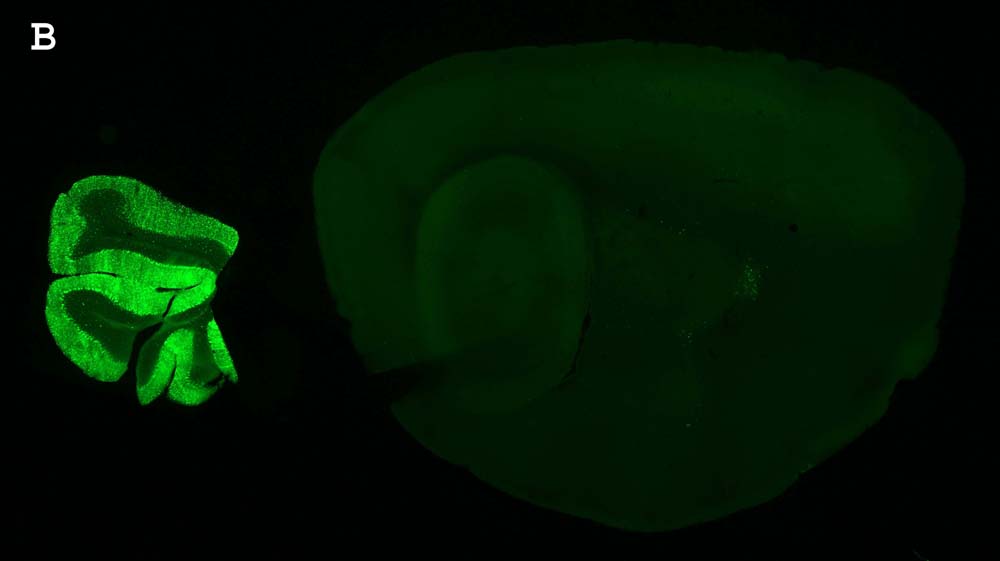


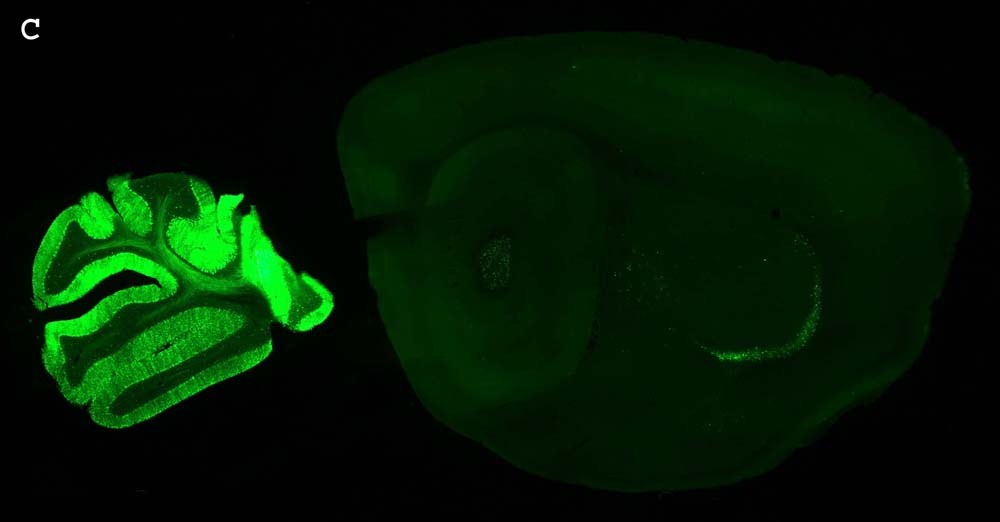


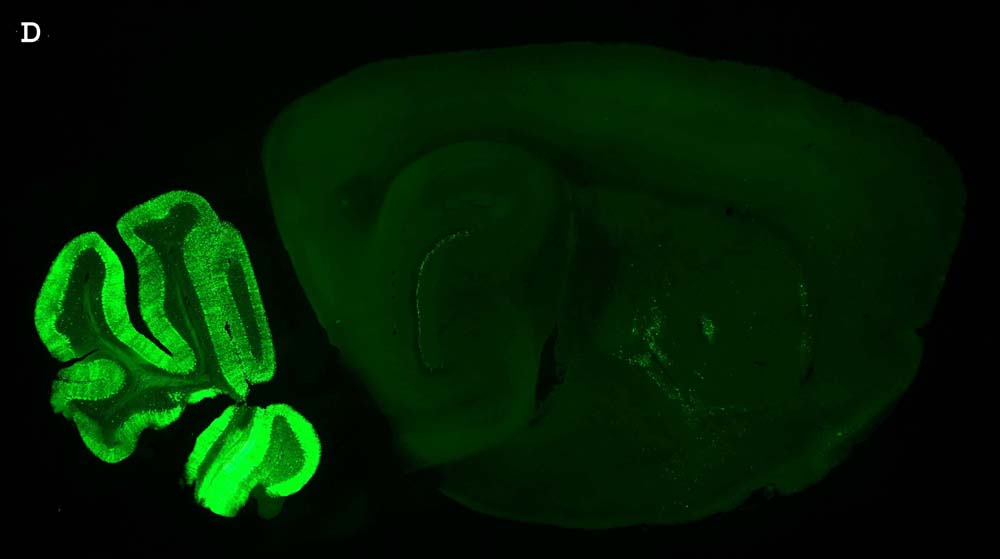


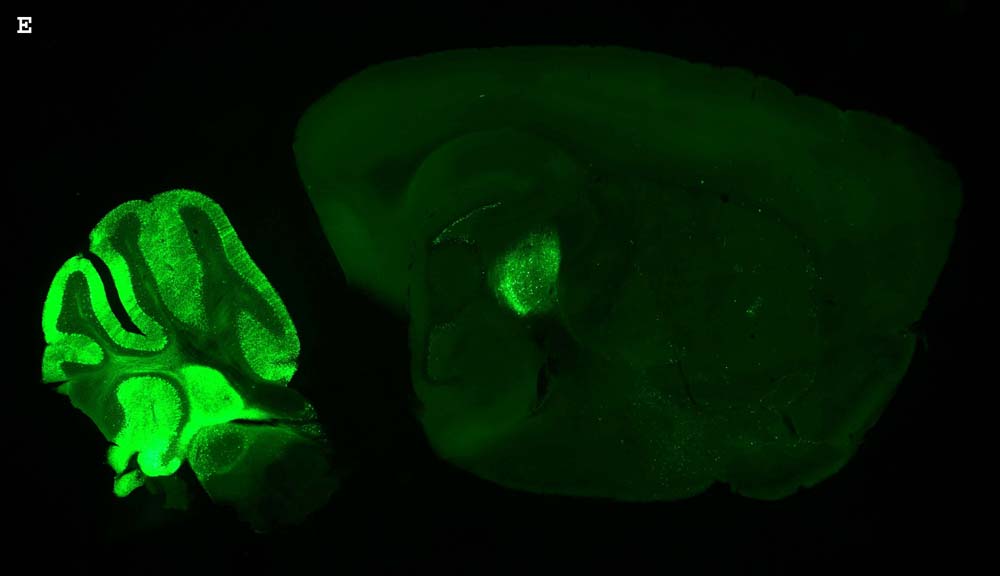


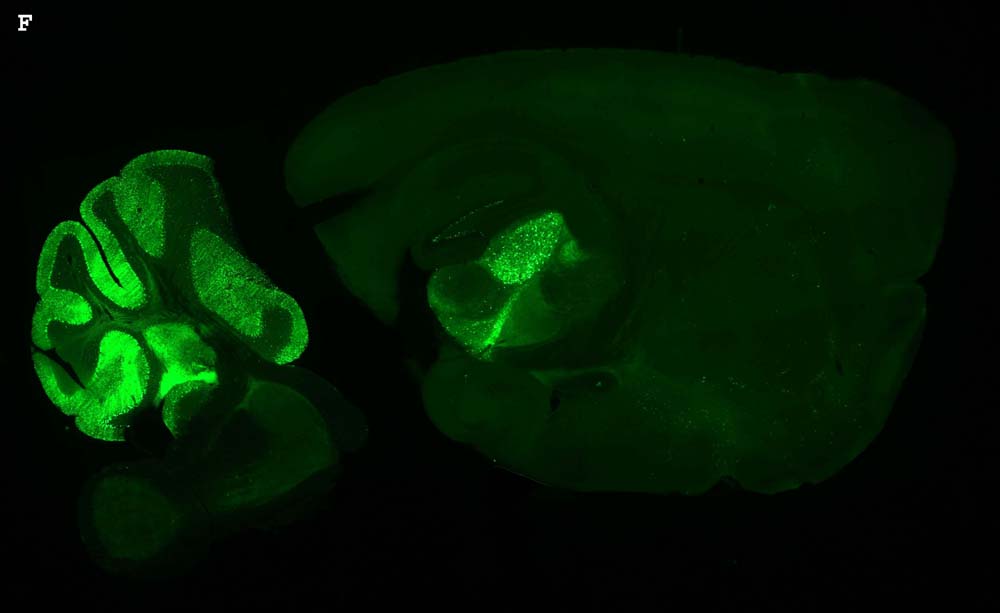


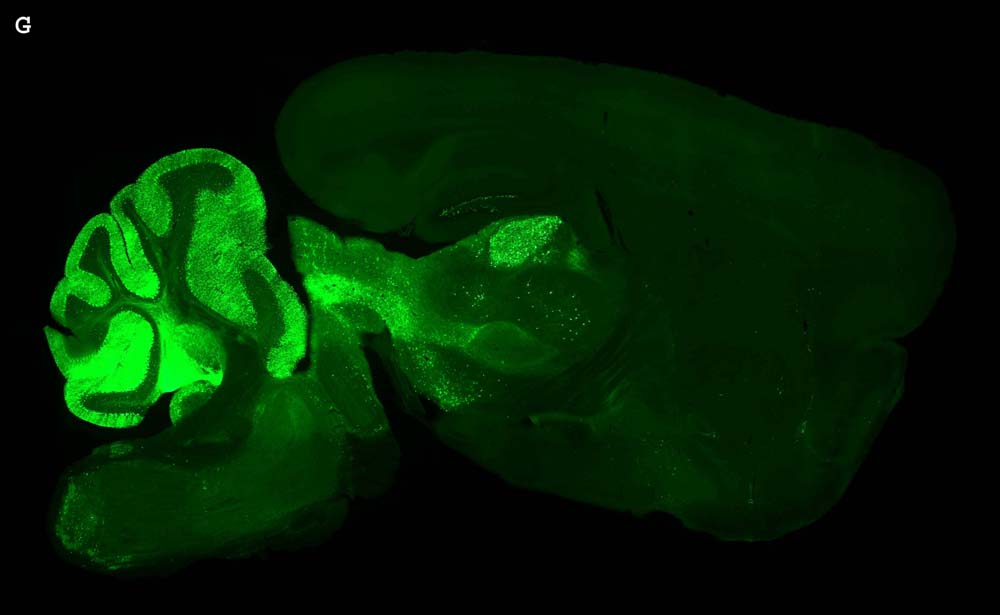


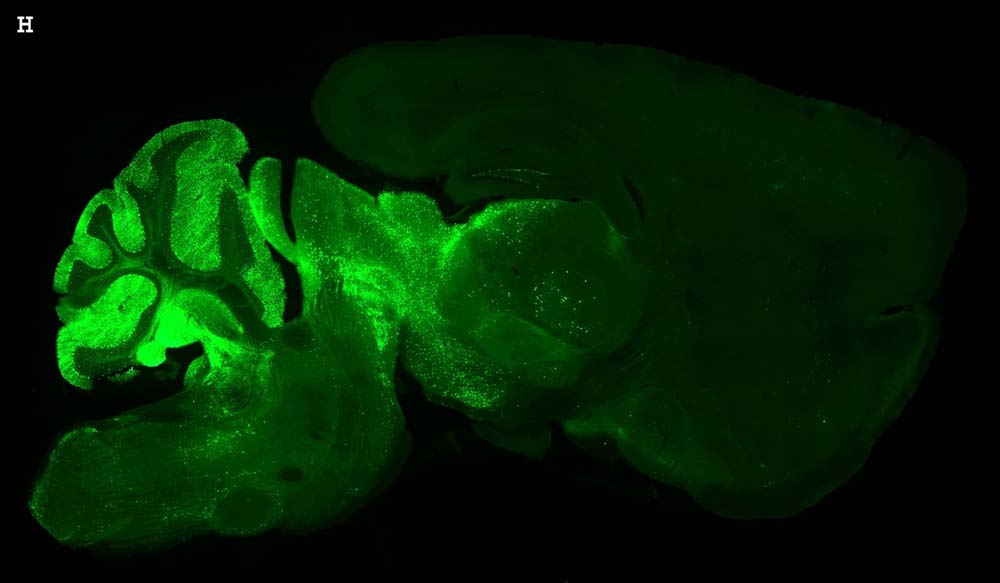


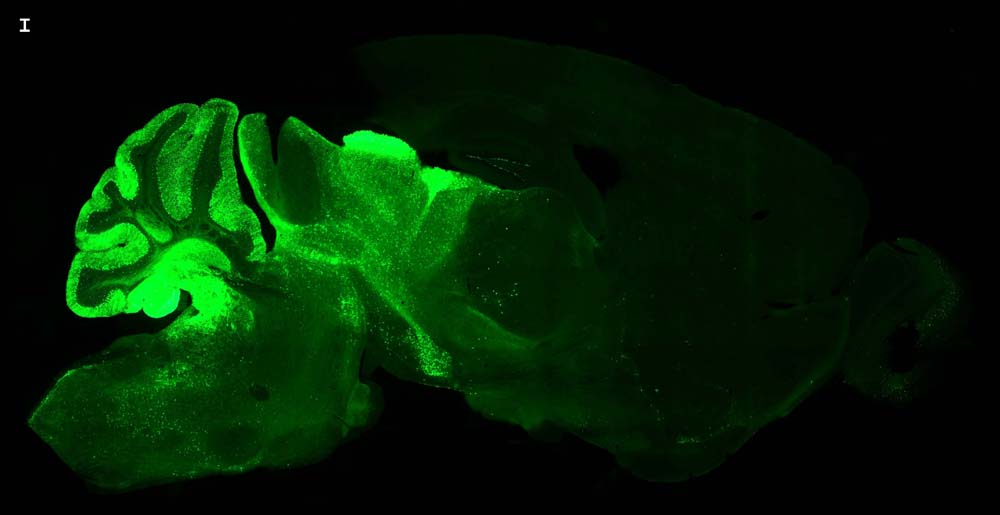


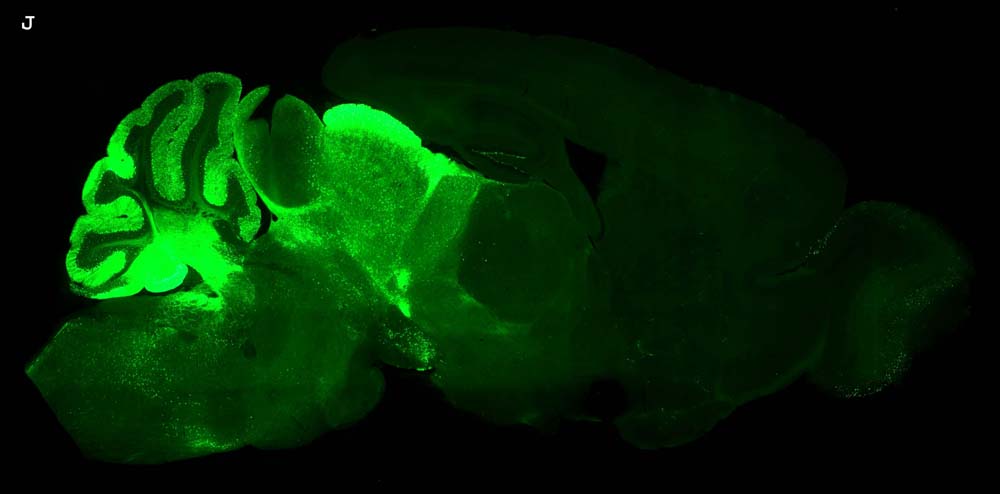


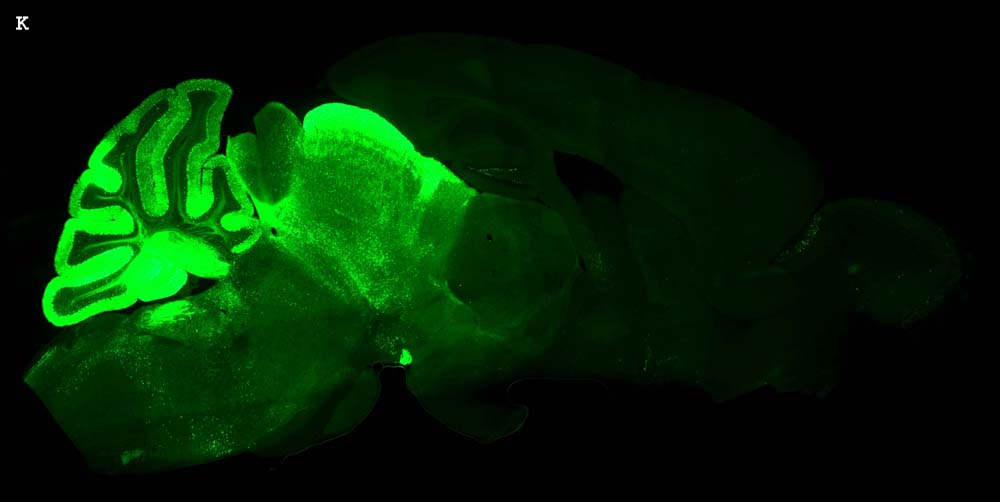

Supplement: Figure S1 — Serial sagittal brain images of GAD67-GFP mouse. A-K. Serial sagittal brain images of eleven sections spaced 300 µm apart. Sections are from a male mouse aged 2.5 months with a C57/BL6 genetic background. Whole brain montages were generated with a Zeiss motorized stage. (0.43 MB DOC) [file pone.0012506.s002.doc]

**Figure S2. Developmental changes of GFP+ neurons**

**
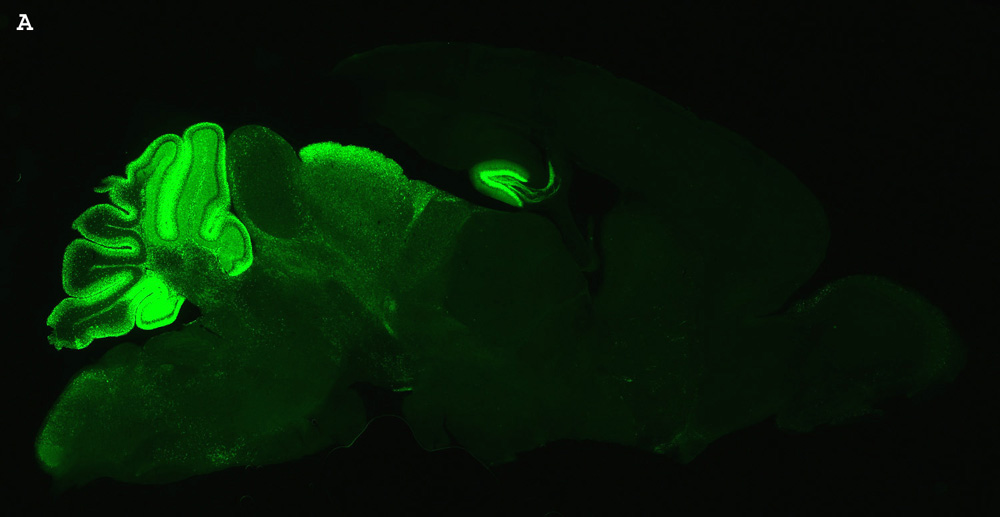
**

**
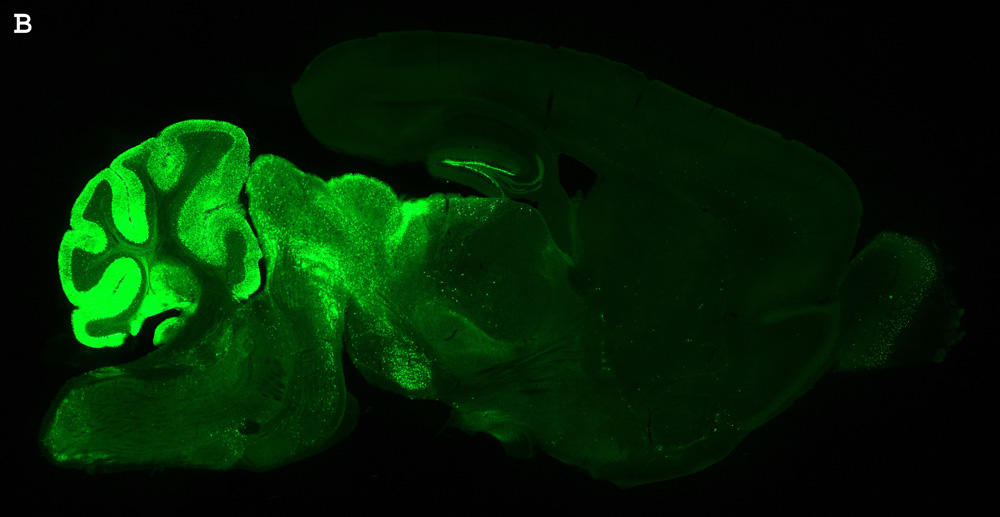
**

**
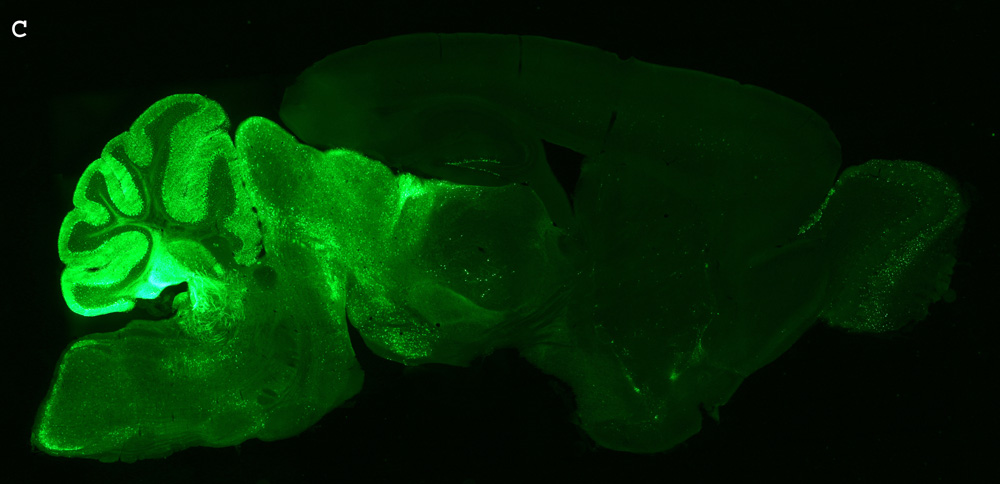
**

**
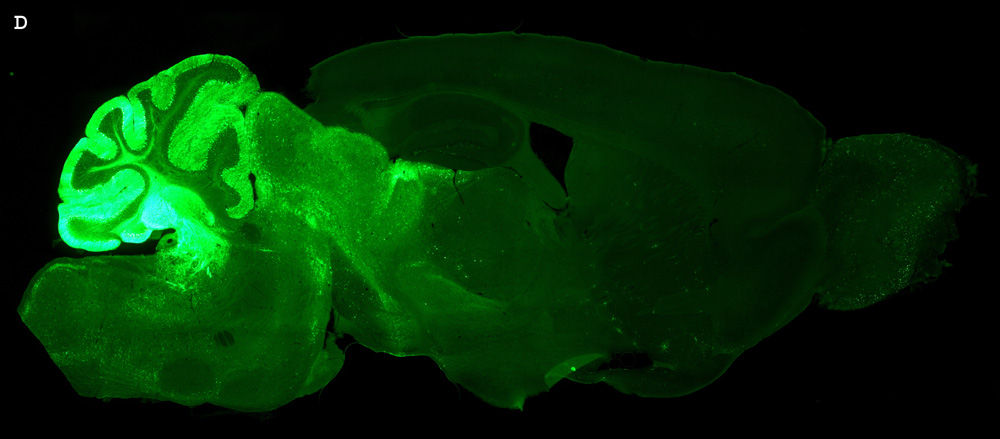
**

Supplement: Figure S2 — The developmental changes of GFP+ neurons. A-D. Whole mouse brain images from GAD67-GFP mice at the age of two weeks (A), one month (B), three months (C) and nine months (D). 50 µm sections were from a male mouse with a C57/BL6 genetic background. Whole brain montages were generated with a Zeiss motorized stage. (0.50 MB DOC) [file pone.0012506.s003.doc]

**Figure S3. GFP+ cells in olfactory bulb are not newborn neurons**


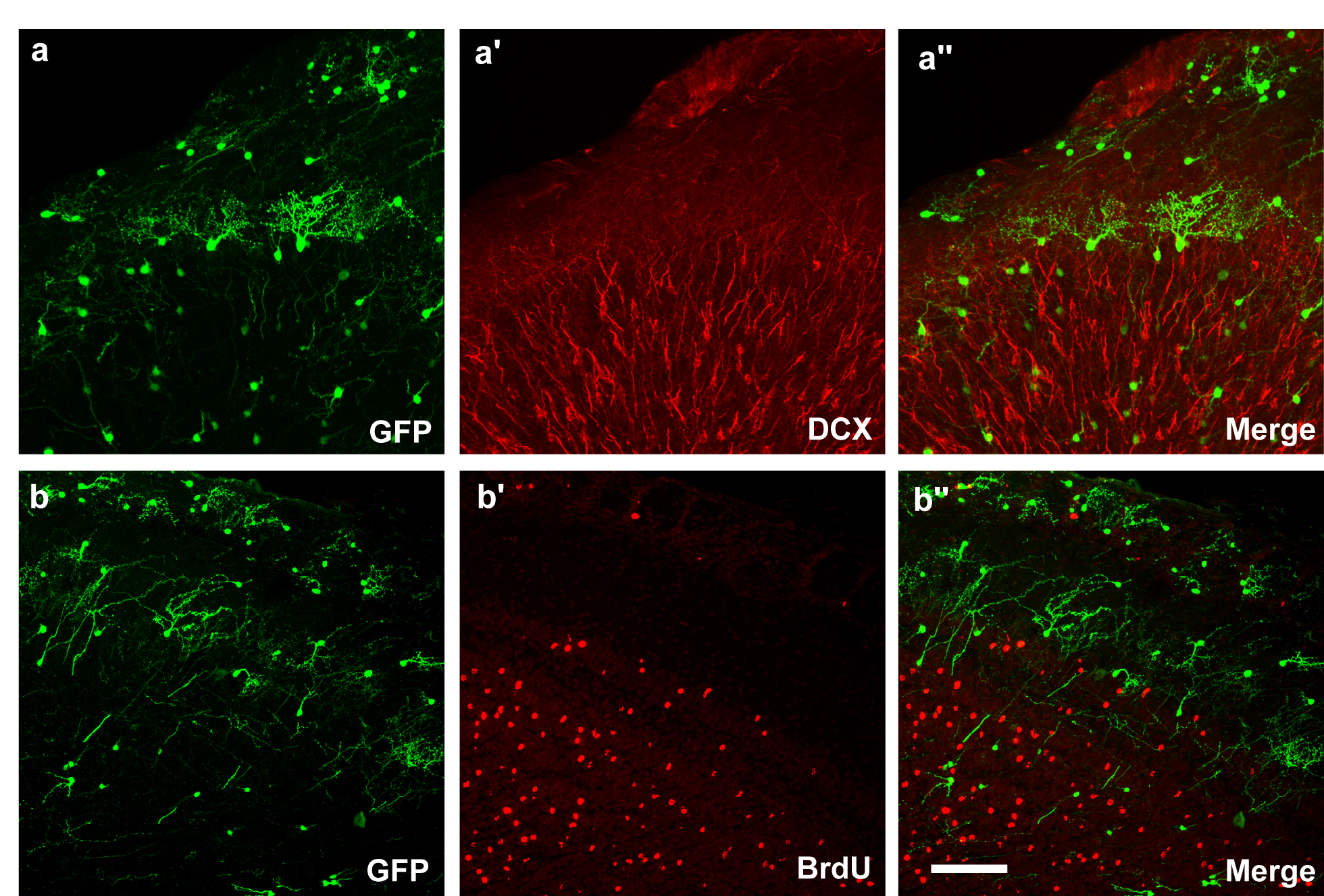

Supplement: Figure S3 — GFP+ cells in olfactory bulb are not newborn neurons. a-b'. Brain sections from a 2-3 month old mouse were stained with GFP (a, b), doublecortin (DCX) (a') and BrdU (b'). a' and b' show the merged images. Images were taken with Olympus FV-1000 confocal microscope using a 20× objective. Scale bar is 100 µm. Neither of doublecortin staining (a') or BrdU labeling (b') colocalized with GFP+ cells in the olfactory bulb. (3.55 MB DOC) [file pone.0012506.s004.doc]
