# Supplementary material for: Fluorescent Labeling of Newborn Dentate Granule Cells in GAD67-GFP Transgenic Mice: A Genetic Tool for the Study of Adult Neurogenesis
Source: PLoS One. 2010 Sep 2;5(9):e12506. doi: 10.1371/journal.pone.0012506 (PMC2932690; doi:10.1371/journal.pone.0012506)

**Figure S4. Dendritic quantification of dentate granule neurons from Thy1-GFP mice and GAD67-GFP mice.**


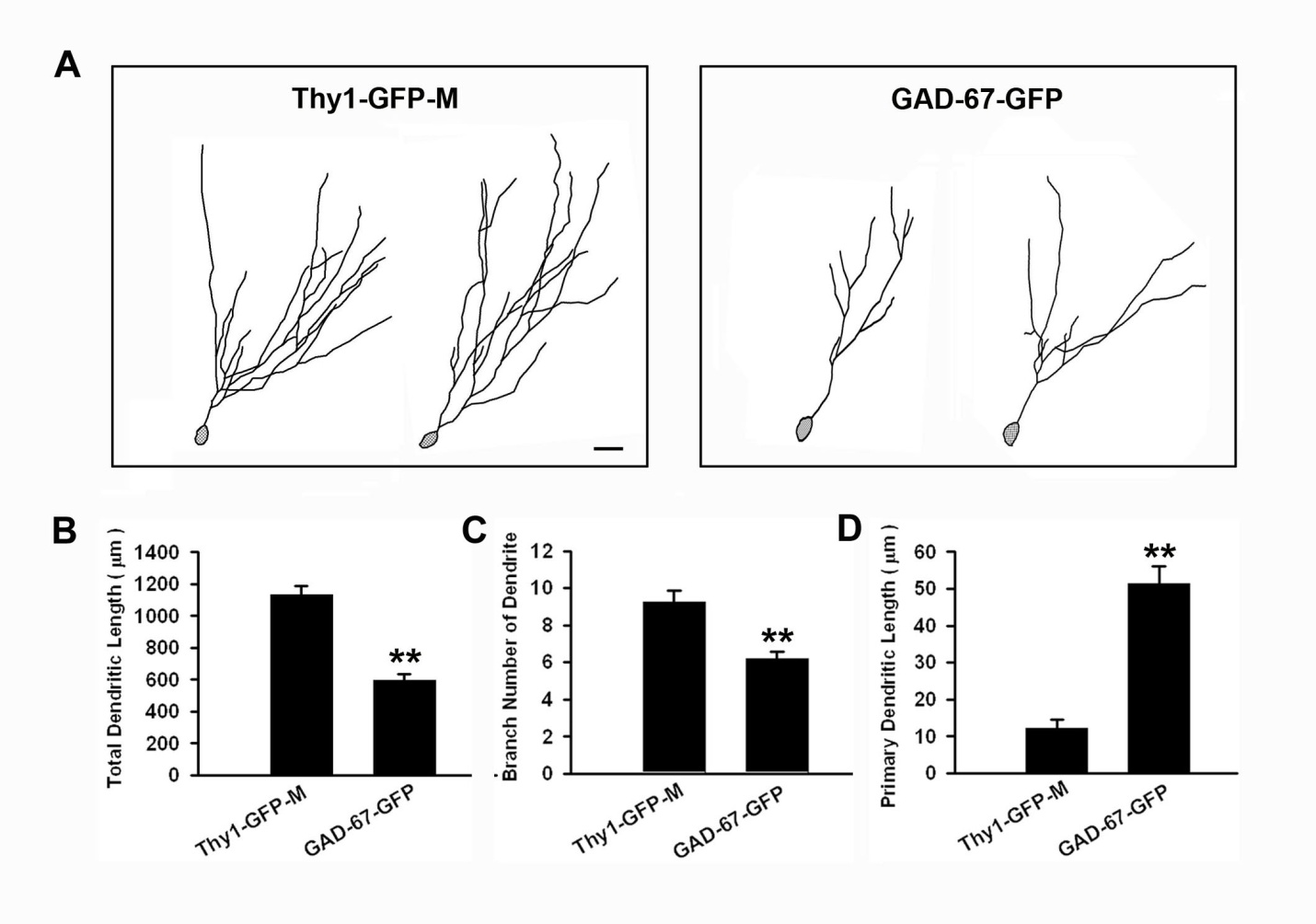

Supplement: Figure S4 — Dendritic quantification of dentate granule neurons from Thy1-GFP mice and GAD67-GFP mice. Thy1-GFP-M line and GAD67-GFP line mice around 7-month-old were paired. For a better quantification, anti-GFP antibody was used to enhance the signal as described above. GFP fluorescence was imaged with an Olympus BX61WI confocal microscope, and dendritic branch numbers and dendritic length were quantified with Nurolucida 9 software (MBF Bioscience). A. Representative examples of dentate granule neurons in Thy1-GFP-M and GAD67-GFP transgenic mice. Scale bar is 20 µm. B-D. Quantitative analysis of total dendritic length (B), branch number (C), and primary dendritic length (D) of dentate granule neurons in Thy1-GFP-M and GAD67-GFP transgenic mice. GAD67-GFP labeled newborn neurons show significantly fewer dendritic branches (6.2±0.35 µm vs. 9.25±0.60 µm, p<0.001) and shorter total dendritic length (595.51±37.02 µm vs. 1131.36±57.02, p<0.001) than Thy1-GFP labeled mature dentate granule neurons. However, they show longer primary dendrites than Thy1-GFP positive mature granule cells (51.39±4.70 µm vs. 12.11±2.29 µm, p<0.001). Data represent Mean±SEM; n = 20 for each group (** p<0.001, student t test). (0.19 MB DOC) [file pone.0012506.s005.doc]
